# Biochemistry, hormones and adipocytokines in prepubertal children born with IUGR evoke metabolic, hepatic and renal derangements

**DOI:** 10.1038/s41598-018-34075-6

**Published:** 2018-10-24

**Authors:** Elpida J. Sidiropoulou, George Paltoglou, George Valsamakis, Alexandra Margeli, Aimilia Mantzou, Ioannis Papassotiriou, Dimitrios Hassiakos, Nicoletta Iacovidou, George Mastorakos

**Affiliations:** 1 Endocrine Unit, “Aretaieion” Hospital, National and Kapodistrian University of Athens, Faculty of Medicine, Athens, Greece; 2 First Department of Pediatrics, “Aghia Sophia” Children’s Hospital, National and Kapodistrian University of Athens, Faculty of Medicine, Athens, Greece; 3 Department of Clinical Biochemistry, “Aghia Sophia” Children’s Hospital, Athens, Greece; 4 Second Department of Obstetrics and Gynecology, “Aretaieion” Hospital, National and Kapodistrian University of Athens, Faculty of Medicine, Athens, Greece; 5 Neonatal Department, “Aretaieion” Hospital, National and Kapodistrian University of Athens, Faculty of Medicine, Athens, Greece

## Abstract

Children born with IUGR develop features of the metabolic syndrome and exhibit deranged markers of hepatorenal physiology. Metabolic and hepatorenal biochemistry and the *rs9939609* FTO polymorphism were investigated in prepubertal children born with IUGR. Ninety-eight prepubertal children (46 IUGR and 52 AGA), subdivided in <5 years and >5 years old groups were included. Anthropometry; creatinine, eGFR, urea, AST, ALT, triglycerides, uric acid, total cholesterol, HDL-c, LDL-c, glucose, C-peptide, insulin and glucagon z-scores; HOMA-IR; leptin and adiponectin concentrations; *rs9939609* FTO polymorphism frequency were measured. In males, weight and ALT were higher and adiponectin was lower, in IUGR < 5 years; C-peptide, insulin and leptin were higher in IUGR > 5 years; C-peptide was higher in all IUGR, than the respective AGA. In females, creatinine and triglycerides were higher in IUGR < 5 years old; creatinine was higher and eGFR was lower in all IUGR, than the respective AGA. In males and females, creatinine was higher in all IUGR, than the respective AGA; C-peptide, insulin and HOMA-IR were lower, and AST was higher in IUGR < 5 than in IUGR > 5 years old. FTO rs9939609 frequency did not differ between IUGR and AGA. In conclusion prepubertal males born with IUGR increased weight, insulin and leptin and decreased adiponectin, as compared to males born AGA, emerge as early metabolic syndrome characteristics. The concentrations of these hormones do not differ between prepubertal males and females born with IUGR. Weight control, healthy nutrition and physical exercise should be recommended to these children. The deranged renal (particularly evident in females below the age of 5) and liver biochemistry in prepubertal children born with IUGR suggests that hepatorenal derangements might commence *in utero*. Regular checkup of biochemical and lipid profile is recommended for all children born with IUGR.

## Introduction

In intrauterine growth restriction (IUGR) fetus fails to achieve its growth potential due to genetic or environmental factors *in utero*^1^. Intrauterine growth restriction is defined as estimated birth weight below the 3rd centile^2,3^. The term small for gestational age (SGA) refers to an infant whose birth weight is below the 10th centile for the appropriate gestational age^2^. This clinical problem affects approximately 8% of pregnancies and is associated with perinatal mortality and morbidity^4^. Several risk factors of maternal, fetal and placental origin have been associated with the development of IUGR^4,5^. Children born with IUGR exhibit catch-up growth during their first months of life^6^. After birth, babies born with IUGR display abnormal biochemical profiles^6,7^.

Intrauterine growth restriction, as originally been suggested in the “thrifty phenotype” hypothesis by Barker *et al*., has been causally associated with the development of metabolic syndrome in adulthood and with long-term metabolic sequelae such as obesity, impaired glucose tolerance, diabetes mellitus type 2 (T2DM), hypertension, dyslipidemia, cardiovascular disease and polycystic ovary syndrome (PCOS)^7–11^. In a rat model, catch-up growth in IUGR was associated with the development of an insulin resistant phenotype of adipose tissue which in its turn contributes to the development of obesity^12^. Girls born with low birth weight, followed by postnatal catch up growth, exhibit increased visceral fat and insulin resistance in pre-school age^11^. Young adults born SGA demonstrate hyperinsulinemia^13^. Furthermore, animal studies demonstrated the association between low birth weight and reduced number of nephrons^14^. Preterm infants with extremely low birth weight exhibit markedly decreased glomerulogenesis^15^. In low birthweight preterm infants, active glomerulogenesis occurs in postnatal period, but it does not suffice to compensate the decrease of the number of nephrons *in utero*^16^. Children born with IUGR are reported to have fewer nephrons but the same glomerular volume, whereas children with birth weight less than 2.5 kg have fewer nephrons and larger glomerular volume than children of higher birth weight^17,18^.

Intrauterine growth restriction has been associated with the development of obesity in the future^7,9^. Obesity in childhood and adulthood has emerged as one of the most serious public health concerns^19,20^. The *rs9939609* polymorphism of the human fat mass and obesity associated (FTO) gene was the first robust identification of a common gene variant to be associated with increased body mass index (BMI) and obesity^21^. In other studies, FTO *rs9939609* polymorphism was associated with insulin resistance, T2DM, metabolic syndrome and cardiovascular disease^22–24^.

To investigate the early development of features of metabolic syndrome as well as the biochemical, hormonal and adipocytokines profiles in prepubertal children born with IUGR a cohort of prepubertal children born with IUGR was studied cross-sectionally for anthropometric, biochemical, hormonal and adipocytokines parameters in comparison with a cohort of prepubertal healthy children born appropriate for gestational age (AGA). The possible association of the *rs9939609* polymorphism of FTO gene with the development of obesity was also investigated.

## Subjects and Methods

### Subjects

Ninety-eight healthy Caucasian children from a Pediatric Endocrinology Center were enrolled in this cross-sectional study [46 (17 male, 29 female; median age: 3.750 years) born full-term IUGR and 52 (26 male, 26 female; median age: 5.125 years) born full-term AGA]. Both IUGR and AGA groups were homogenous by age when a statistical comparison of the means with Factors ANOVA followed by an LSD post-hoc test was performed (Table 1). Children were characterized as IUGR or AGA when their birth weight was below the 3rd customized centile or between the 10^th^ and 90^th^ customized centile for gestational age, respectively. Children between the 3^rd^ and 10^th^ customized centile were excluded. The classification of IUGR and AGA was done according to the Fenton 2013 growth charts^25^.

**Table 1:** Comparison of age between male and female children born IUGR and AGA at the observation time.

	Male (n = 43)	Female (n = 55)		
Mean Age ± SE	5.20 ± 0.46	4.44 ± 0.38		
Median Age ± SD	5.25 ± 3.03	3.67 ± 2.83		
	**IUGR** **(n** = **17)**	**AGA** **(n** = **26)**	**IUGR** **(n** = **29)**	**AGA** **(n** = **26)**
Mean Age ± SE	5.02 ± 0.58	5.32 ± 0.67	3.8 ± 0.46	5.16 ± 0.60
Median Age ± SD	4.33 ± 2.41	5.29 ± 3.41	3.50 ± 2.49	4.54 ± 3.06

Statistical comparisons were performed with multiple factors ANOVA followed by an LSD post-hoc test for the means and a non-parametric median test for the medians. Neither analysis revealed any statistical differences among the groups.

All participants were prepubertal (Tanner I) and of normal weight (Table 2). The male to female ratio for all subjects taken as a whole was 43/55 = 0.78; for the children born with IUGR was 17/29 = 0.58 and for the children born AGA was 26/26 = 1. When these figures were tabulated in a 2 × 2 contingency table, the calculated Pearson’s chi-square test was 1.686 with a p-value 0.194, which is not significant at p < 0.05, indicating that the male to female ratio between the IUGR and the AGA groups did not differ significantly. Both IUGR and AGA were divided in 8 groups according to their gender and age (males born IUGR < 5 years old; males born AGA < 5years old; males born IUGR > 5 years old; males born AGA > 5 years old; females born IUGR < 5 years old; females born AGA < 5 years old; females born IUGR > 5 years old; females born AGA > 5 years old). The cut-off point of 5 years represents the lower limit of initiation of adrenarche. Thus, it was chosen to discriminate for differences possibly related to adrenal steroid hormones increase in adrenarche^26^. As previously reported, the concentrations of the adrenal androgen DHEAS in peripheral circulation were maintained at a minimum level until the age of 5 years in both male and female children, after which a gradual increase was observed^27^. The Institutional Review Board of the National and Kapodistrian University of Athens approved the study which was conducted in accordance with the Declaration of Helsinki on human experimentation as revised in 1996. Informed written consent was obtained from the parent/guardians of each child.

**Table 2:** Comparison of anthropometric parameters between children born IUGR and AGA at birth and at the observation time expressed as mean ± S.E. Statistical comparisons were performed with multiple factors ANOVA.

	IUGR(n = 46)	AGA(n = 52)
Birth weight(g)	2272.56 ± 60.03*	2965.10 ± 57.09
Birth length(cm)	46.61 ± 0.46*	50.03 ± 0.25
Waist/hip ratio	0.90 ± 0.01	0.91 ± 0.01
Weight z-score	0.39 ± 0.21	0.68 ± 0.18
Height z-score	0.13 ± 0.20	0.54 ± 0.21
BMI z-score	0.37 ± 0.25	0.56 ± 0.15

* denotes statistically significant difference from the respective AGA group.

### Protocol

At the first visit, all children had an initial clinical examination by the same physician (E.S.). Their birth weight and length were recorded (Tables 2 and 3). Anthropometric measurements were recorded (the mean value of two measurements for each subject) and a venous blood sampling was performed at fasting. Body weight was measured to the nearest 0.1 kg (Beam Balance 710, Seca, Birmingham, UK), while children were wearing their underclothes. Height was measured to the nearest 0.1 cm using a stadiometer (Seca 208, Hanover, MD), while children were barefoot. BMI (in kilograms per square meter) was calculated. Weight, height and BMI z-scores were calculated from the respective data for the Greek population (Tables 2 and 3)^28^. Maximum hip and waist circumferences were measured in cm in duplicate with a flexible tape and waist-to-hip ratio was calculated (Table 2). Blood was collected into tubes containing serum separating (SST)-gel and was centrifuged for serum separation. Serum was immediately analyzed after sample collection, for biochemical and hormonal parameters determination, while the remaining serum samples were stored frozen in multiple aliquots (at −80 °C) for further analyses. Serum samples were thawed once before analysis and were protected from light and auto-oxidation. All assays were performed in duplicate and the mean value was recorded.

**Table 3:** Comparison of anthropometric parameters between children born IUGR and AGA at birth (birth weight and length) and at the observation time (weight z-score, height z-score, BMI z-score) expressed as mean ± S.E. Statistical comparisons were performed with multiple factors ANOVA.

	Male (n = 43)				Female (n = 55)			
	IUGR (n = 17)		AGA (n = 26)		IUGR (n = 29)		AGA (n = 26)	
Weight z-score	1.14 ± 0.41^+^		0.70 ± 0.30		−0.045 ± 0.18*		0.65 ± 0.21	
Height z-score	0.57 ± 0.33		0.65 ± 0.31		−0.12 ± 0.25		0.42 ± 0.28	
BMI z-score	1.10 ± 0.41^+^		0.50 ± 0.23		−0.058 ± 0.29		0.61 ± 0.19	
	**Below 5 years**		**Above 5 years**		**Below 5 years**		**Above 5 years**	
	**IUGR** **(n** = **9)**	**AGA** **(n** = **14)**	**IUGR** **(n** = **8)**	**AGA** **(n** = **12)**	**IUGR** **(n** = **21)**	**AGA** **(n** = **14)**	**IUGR** **(n** = **8)**	**AGA** **(n** = **12)**
Birth weight(g)	2286.43 ± 92.50*	2958.00 ± 110.62	2396.25 ± 83.45*	2977.14 ± 120.83	2191.19 ± 104.63*	2990.83 ± 135.14	2361.43 ± 141.70*	2931.25 ± 93.70
Birth Length(cm)	46.43 ± 1.05*	49.75 ± 0.67	48.17 ± 0.95*	50.04 ± 0,48	45.85 ± 0.70*	50.05 ± 0.59	47.64 ± 0.86*	50.26 ± 0.29
Weight z-score	0.50 ± 0.36^#,^*	0.11 ± 0.29^#^	1.86 ± 0.72	1.25 ± 0.47	−0.26 ± 0.19	0.47 ± 0.14	0.52 ± 0.39	0.84 ± 0.41
Height z-score	0.074 ± 0.39^+^	0.17 ± 0.43	1.13 ± 0.48	1.06 ± 0.43	0.006 ± 0.28	0.04 ± 0.32	−0.46 ± 0.55	0.80 ± 0.45
BMI z-score	0.63 ± 0.51^#^	0.03 ± 0.24	1.63 ± 0.65	0.91 ± 0.34	−0.39 ± 0.30*	0.61 ± 0.23	0.83 ± 0.66	0.61 ± 0.32

* denotes statistically significant difference from the respective AGA group.

^#^ denotes statistically significant difference from the respective group aged >5 years old.

^+^ denotes statistically significant difference from the respective female group.

### Assays

Biochemical parameters including serum glucose, total cholesterol, high density lipoprotein HDL- and low density lipoprotein LDL- cholesterol (HDL-c and LDL-c, respectively), triglycerides (TG), uric acid, creatinine, urea, aspartate aminotransferase (AST), alanine aminotransferase (ALT) were determined by using the Siemens Advia 1800 Clinical Chemistry Analyzer (Siemens Healthcare Diagnostics, Tarrytown, NY, USA).

Glomerular Filtration Rate (GFR) was estimated according to the Bedside Schwartz formula (2009): eGFR = 0.413 × height (cm)/creatinine (mg/dL)^29^.

Serum insulin concentrations were measured with an electrochemiluminescence immunoassay using the automated analyser Cobas e411 and the Elecsys Insulin Kit (Roche Diagnostics, Basel, CH); serum C-peptide concentrations were measured with a solid phase two-site chemiluminescent immunometric assay using a chemiluminescence autoanalyzer Immulite 2000 (Siemens Healthcare Diagnostics, UK); serum glucagon concentrations were measured with a radioimmuoassay kit (Euro-Diagnostica, Malmo, Sweden); serum leptin concentrations were determined by using a Milliplex Map Human Metabolic Panel (Millipore, USA), which is based on the Luminex xMAP technology; serum adiponectin concentrations were determined by using an enzyme-linked immunoabsorbent assay (ELISA) purchased from Orgenium Laboratories, Finland. The intra- and inter- assay coefficients of variation (CV) and the sensitivity limits of these assays were, respectively: 2.0%, 2.8% and 0.2 μU/ml; 3.5%, 6.2% and 0.09 ng/ml; 2.5%, 8.8% and 3 pmol/L; <7%, <10% and 0.184 ng/ml; <10%, <12% and 0.185 ng/ml.

### Indices of carbohydrate metabolism

Insulin resistance was estimated by the homeostasis model assessment (HOMA-IR) = Glucose × Insulin/405 using fasting concentrations (glucose and insulin in mg/dl and mU/L, respectively)^30^.

Glucose-to-insulin ratio (G/I) was calculated using fasting concentrations (glucose and insulin in mg/dl and mU/L, respectively)^31^.

The quantitative insulin sensitivity check index (QUICKI) was calculated as follows: 1/[log(fasting insulin μU/mL) + log(fasting glucose mg/dl)^32^.

β-cell secretion of insulin was estimated by the HOMA-β = [(360 × Insulin)/(Glucose-63)]% using fasting concentrations (glucose and insulin in mg/dl and mU/L, respectively)^33^.

### FTO Genotyping

Genotyping of FTO was performed as previously described^34^. DNA was extracted from whole blood samples using the Qiamp Blood Kit (Qiagen, Germany) and then it was stored at −20 °C until analysis. The FTO single nucleotide polymorphism (SNP) (*rs9939609*) was genotyped by polymerase chain reaction (PCR) and restriction fragment length polymorphism analyses. Genomic deoxyribonucleic acid (DNA) (20 ng) was incubated in a 10 μl solution containing 1 Χ NH_4_ buffer, 2.5 mmol/L Mg, 200 μmol/L each deoxyribose nucleoside triphosphate (dNTP), 20 pmol forward (5-AACTGGCTCTTGAATGAAATAGGATTCAGA-3) and reverse (5-AGAGTAACAGAGACTATCCAAGTGCAGTAC-3) oligonucleotide primers, and 0.5 U *Taq* DNA polymerase (Bioline Ltd., London, UK). The PCR mix was incubated at 94 °C for 5 min followed by 20 cycles of 94 °C for 45 sec, 61 °C for 45 sec, and 72 °C for 45 sec. After this, the PCR mix was incubated for 15 cycles of 94 °C for 45 sec, 51 °C for 45 sec, and 72 °C for 45 sec, followed by a final incubation at 72 °C for 10 min. Then it was incubated at 37 °C for 16 h with 2 U *ScaI* (New England Biolabs, Hitchin, UK). Upon running the final products on a 3% agarose gel, the wild-type T allele produced a 182-bp band and the polymorphic A allele produced 154- and 28-bp bands.

### Statistical analysis

Results are reported as mean ± standard error (SE) or median and interquartile range (25^th^–75^th^ percentile). Statistical significance was set at p < 0.05. Standard deviations scores (z-scores) of biochemical and glucose metabolism parameters were calculated from the respective mean and SD values for age from data of our laboratory. Anthropometric measurements, biochemical parameters, glucose metabolism parameters and adipocytokines were compared between IUGR and AGA groups by employing multiple factors analysis of variance (ANOVA). Significant main effects were revealed by Fischer’s *post-hoc* test.

Comparison of the frequencies of the FTO genotypes in IUGR and AGA children for each polymorphism was performed with a 2 × 3 Pearson’s chi-square (3 genotypes for the 2 studied groups) test as previously described^35^. All polymorphisms were tested for Hardy-Weinberg-Equilibrium (HWE)^35^. Allele frequencies comparison was also performed in the same way (wild-type T allele versus polymorphic A allele for the two studied groups)^35^. To test the hypothesis that carrying the polymorphic A allele increased the risk for IUGR as compared to AGA (dominant model), the genotypes containing the polymorphic allele were pooled in a 2 × 2 contingency table and were compared with a 2 × 2 Pearson’s chi-square test. To test the hypothesis that carrying two polymorphic A alleles increased the risk for IUGR as compared to AGA (recessive model), the genotypes containing the wild-type allele were pooled in a 2 × 2 contingency table followed by a 2 × 2 Pearson’s chi-square test. In the 2 × 2 contingency tables Yates correction was used to prevent overestimation of statistical significance for small data^35^. All statistical analyses were performed with the Statistica 6 software (StatSoft, Tulsa, USA).

## Results

### Anthropometry in children born with IUGR and in children born AGA

Mean birth weight and mean birth length were significantly lower in subjects born with IUGR than in subjects born AGA (p < 0.05) (Table 2). In female subjects mean weight z-score was significantly lower in those born with IUGR than in those born AGA (p < 0.05). In subjects born with IUGR mean weight and BMI z-scores were significantly higher in males than in females (p < 0.05) (Table 3).

In male subjects aged <5 years old mean weight z-score was significantly higher in those born with IUGR than in those born AGA (p < 0.05). In male subjects born with IUGR mean BMI z-score was significantly lower in those aged <5 than in those aged >5 years old (p < 0.05). In female subjects aged <5 years old mean BMI z-score was significantly lower in those born with IUGR than in those born AGA (p < 0.05). In subjects born with IUGR and aged <5 years old mean height z-score was significantly higher in males than in females (p < 0.05) (Table 3).

### Biochemical parameters in children born with IUGR and in children born AGA

The measurements of biochemical parameters in the studied groups at the observation time, expressed in z-scores, are presented in Table 4.

**Table 4:** Comparison of biochemical parameters between children born IUGR and AGA at the observation time expressed as mean ± S.E.

	Male (n = 43)				Female (n = 55)			
	IUGR (n = 17)		AGA (n = 26)		IUGR (n = 29)		AGA (n = 26)	
Creatinine z-score	0.33 ± 0.24*		−0.29±0.21		0.36 ± 0.11*		−0.35 ± 0.22	
Urea z-score	0.26 ± 0.19		0.04 ± 0.17		−0.14 ± 0.22		−0.06 ± 0.21	
AST z-score	0.04 ± 0.25		−0.09 ± 0.19		0.32 ± 0.20*		−0.31 ± 0.17	
ALT z-score	0.55 ± 0.34^+^		0.05 ± 0.21		−0.13 ± 0.14		−0.26 ± 0.17	
Triglycerides z-score	−0.31 ± 0.25		−0.23 ± 0.15^+^		0.49 ± 0.20*		−0.13 ± 0.18	
Uric acid z-score	0.01 ± 0.25		−0.00 ± 0.20		0.10 ± 0.19		−0.13 ± 0.21	
Total chol z-score	−0.05 ± 0.22		−0.15 ± 0.21		0.31 ± 0.17		−0.17 ± 0.21	
HDL-c z-score	0.32 ± 0.28		0.09 ± 0.18		−0.04 ± 0.17		−0.27 ± 0.20	
LDL-c z-score	−0.12 ± 0.18		−0.16 ± 0.20		0.35 ± 0.18		−0.16 ± 0.21	
	**Below 5 years**		**Above 5 years**		**Below 5 years**		**Above 5 years**	
	**IUGR** **(n** = **9)**	**AGA** **(n** = **14)**	**IUGR** **(n** = **8)**	**AGA** **(n** = **12)**	**IUGR** **(n** = **21)**	**AGA** **(n** = **14)**	**IUGR** **(n** = **8)**	**AGA** **(n** = **12)**
Creatinine z-score	0.37 ± 0.34	−0.22 ± 0.24	0.28 ± 0.37	−0.34 ± 0.34	0.23 ± 0.13*	−0.70 ± 0.31	0.71 ± 0.08	−0.02 ± 0.30
Urea z-score	0.39 ± 0.23	0.05 ± 0.31	0.11 ± 0.32	0.03 ± 0.18	0.01 ± 0.25	−0.06 ± 0.28	−0.59 ± 0.41	−0.06 ± 0.33
AST z-score	0.53 ± 0.24^#,^*	−0.38 ± 0.23	−0.46 ± 0.38	0.15 ± 0.29	0.61 ± 0.23^#,^*	−0.48 ± 0.24	−0.42 ± 0.29	−0.17 ± 0.24
ALT z-score	0.71 ± 0.27*	−0.36 ± 0.28	0.38 ± 0.65	0.37 ± 0.28^+^	0.02 ± 0.16	−0.13 ± 0.30	−0.53 ± 0.23	−0.37 ± 0.18
Triglycerides z-score	−0.69 ± 0.13^+^	−0.08 ± 0.17	0.13 ± 0.47	−0.38 ± 0.24	0.60 ± 0.27*	−0.36 ± 0.18	0.22 ± 0.21	0.09 ± 0.29
Uric acid z-score	−0.28 ± 0.22	0.04 ± 0.33	0.31 ± 0.45	−0.03 ± 0.26	−0.20 ± 0.21^#^	0.04 ± 0.35	0.86 ± 0.20*	−0.27 ± 0.24

Statistical comparisons were performed with multiple factors ANOVA.

* denotes statistically significant difference from the respective AGA group.

^#^ denotes statistically significant difference from the respective group aged >5 years old.

^+^ denotes statistically significant difference from the respective female group.

**Figure 1:**
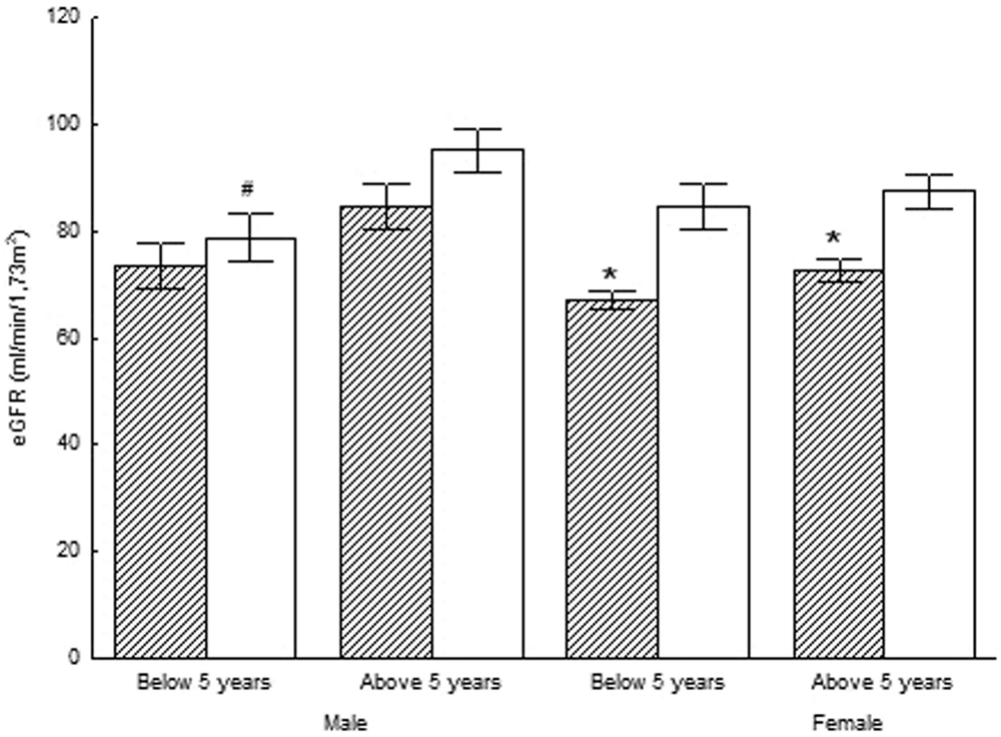
Estimated Glomelural Filtration Rate (eGFR) in the IUGR (hatched bars) and AGA (white bars) groups. Significant differences were assessed by One-Way factors ANOVA (p < 0.05). ^+^ denotes statistically significant difference from the respective female group. * denotes statistically significant difference from the respective AGA group. ^#^ denotes statistically significant difference from the respective group of more than 5 years old age.

Mean creatinine z-score was significantly higher in subjects born with IUGR than in those born AGA (p < 0.05). In female subjects aged <5 years old mean creatinine z-score was significantly higher in subjects born with IUGR than in those born AGA (p < 0.05). In all female subjects mean estimated GFR value was significantly lower in subjects born with IUGR than in those born AGA (p < 0.05) (Fig. 1). Mean urea z-score was not significantly different among the studied groups (p < 0.05).

In subjects born with IUGR mean AST z-score was significantly higher in those aged <5 years old than in those aged >5 years old and also than in subjects born AGA aged <5 years old (p < 0.05). In all female subjects mean AST z-score was significantly higher in those born with IUGR than in those born AGA (p < 0.05). The mean ALT z-score was significantly higher in male subjects born with IUGR and aged <5 years old than in their respective control AGA subjects (p < 0.05). Mean ALT z-score was significantly higher in male subjects born with IUGR than in female subjects born with IUGR (p < 0.05).

Mean total cholesterol, HDL-c and LDL-c z-scores were not significantly different among the studied groups (p < 0.05). In all female subjects mean TG z-score was significantly higher in those born with IUGR than in those born AGA (p < 0.05), while it was significantly higher in female subjects born with IUGR and aged <5 years old than in their respective control AGA subjects (p < 0.05). Mean TG z-score was significantly lower in male subjects born with IUGR and aged <5 years old than in their respective female subjects born with IUGR (p < 0.05).

In female subjects born with IUGR mean z-score of uric acid was significantly lower in those aged <5 than in those aged >5 years old (p < 0.05), while it was significantly higher in female subjects born with IUGR and aged >5 years old than in their respective control AGA subjects (p < 0.05).

### Glucose metabolism parameters and insulin resistance indexes in children born with IUGR and in children born AGA

The measurements of glucose metabolism parameters of the studied groups at the observation time are presented in Table 5. Mean glucose z-score was significantly lower in male born with IUGR and aged <5 years old than in their respective control AGA and than in subjects born with IUGR and aged >5 years old (p < 0.05). Mean glucose z-score was significantly higher in male subjects born with IUGR and aged >5 years old than in their respective female subjects born with IUGR (p < 0.05).

**Table 5:** Comparison of glucose metabolism and hormone parameters between children born IUGR and AGA at the observation time expressed as mean ± S.E.

	Male (n = 43)				Female (n = 55)			
	IUGR (n = 17)		AGA (n = 26)		IUGR (n = 29)		AGA (n = 26)	
Glucose z-score	−0.01 ± 0.30		0.28 ± 0.17		−0.14 ± 0.18		−0.12 ± 0.19	
C-peptide z-score	0.51 ± 0.38*^,+^		−0.20 ± 0.18		−0.15 ± 0.15		−0.02 ± 0.21	
Insulin z-score	0.43 ± 0.29		−0.09 ± 0.18		−0.11 ± 0.17		−0.07 ± 0.19	
Glucagon z-score	0.07 ± 0.23		−0.38 ± 0.13		0.53 ± 0.35		0.03 ± 0.26	
	**Below 5 years**		**Above 5 years**		**Below 5 years**		**Above 5 years**	
	**IUGR** **(n** = **9)**	**AGA** **(n** = **14)**	**IUGR** **(n** = **8)**	**AGA** **(n** = **12)**	**IUGR** **(n** = **21)**	**AGA** **(n** = **14)**	**IUGR** **(n** = **8)**	**AGA** **(n** = **12)**
Glucose z-score	−0.64 ± 0.35^#,^*	0.73 ± 0.23^#,+^	0.71 ± 0.38^+^	−0.13 ± 0.19	−0.10 ± 0.23	−0.12 ± 0.21	−0.27 ± 0.27	−0.12 ± 0.32
C-peptide z-score	−0.27 ± 0.27^#^	0.09 ± 0.40	1.20 ± 0.58^+,^*	−0.39 ± 0.13	−0.15 ± 0.20	−0.05 ± 0.31	−0.16 ± 0.17	−0.00 ± 0.29
Insulin z-score	−0.05 ± 0.33^#^	0.08 ± 0.36	0.98 ± 0.44^+,^*	−0.25 ± 0.12	−0.05 ± 0.23	−0.11 ± 0.17	−0.26 ± 0.23	−0.02 ± 0.34
Glucagon z-score	0.09 ± 0.43	−0.28 ± 0.24	0.04 ± 0.19	−0.44 ± 0.16	0.40 ± 0.26	0.01 ± 0.33	0.75 ± 0.88	0.05 ± 0.40
HOMA-IR	0,79 ± 0,31^#^	1,04 ± 0,48	1,76 ± 0,32	0,97 ± 0,12	0,88 ± 0,22	0,67 ± 0,17	0,78 ± 0,16	1,18 ± 0,24

Statistical comparisons were performed with multiple factors ANOVA.

* denotes statistically significant difference from the respective AGA group.

^#^ denotes statistically significant difference from the respective group aged >5 years old.

^+^ denotes statistically significant difference from the respective female group.

Mean C-peptide z-score was significantly higher in all male subjects born with IUGR than in all male subjects born AGA (p < 0.05). Mean C-peptide and insulin z-scores as well as mean HOMA-IR value were significantly lower in male subjects born with IUGR and aged <5years old than in male subjects born with IUGR and aged >5 years old (p < 0.05). The latter had mean C-peptide and insulin z-scores higher than in their respective male subjects born AGA as well as than in female subjects born with IUGR and aged >5 years old (p < 0.05).

Mean HOMA-β, QUICKI and glucose/insulin ratio values did not differ significantly among the studied groups (p < 0.05) (data not shown).

### Adipocytokines in children born with IUGR and in children born AGA

**Figure 2:**
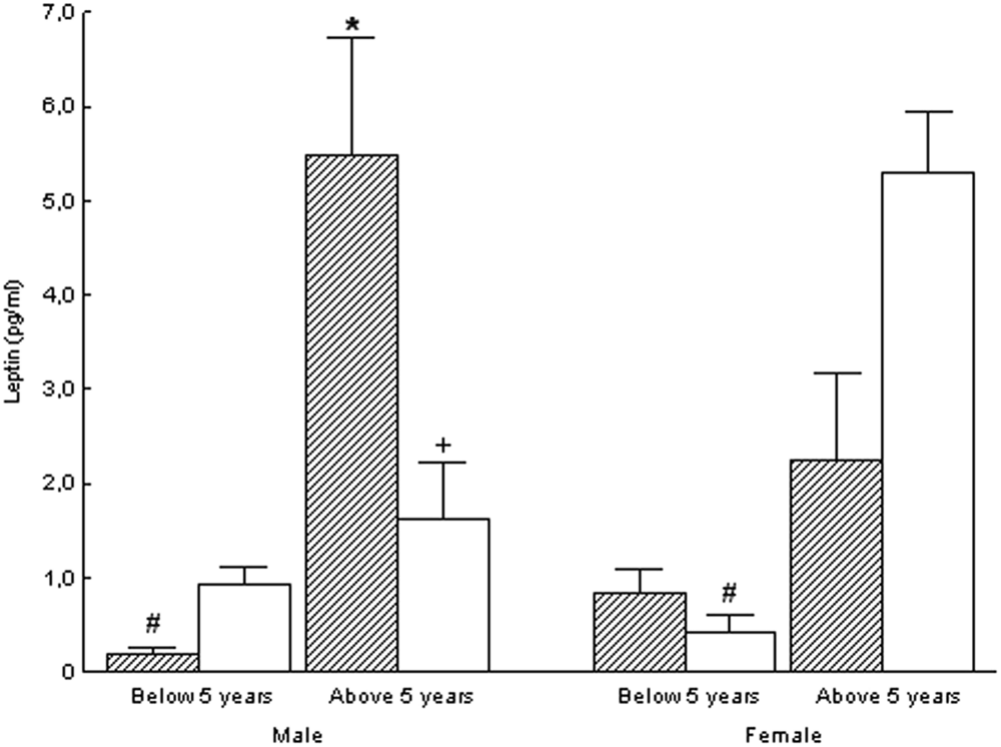
Leptin in the IUGR (hatched bars) and AGA (white bars) groups. Significant differences were assessed by One-Way factors ANOVA (p < 0.05). ^+^ denotes statistically significant difference from the respective female group. * denotes statistically significant difference from the respective AGA group. ^#^ denotes statistically significant difference from the respective group of more than 5 years old age.

**Figure 3:**
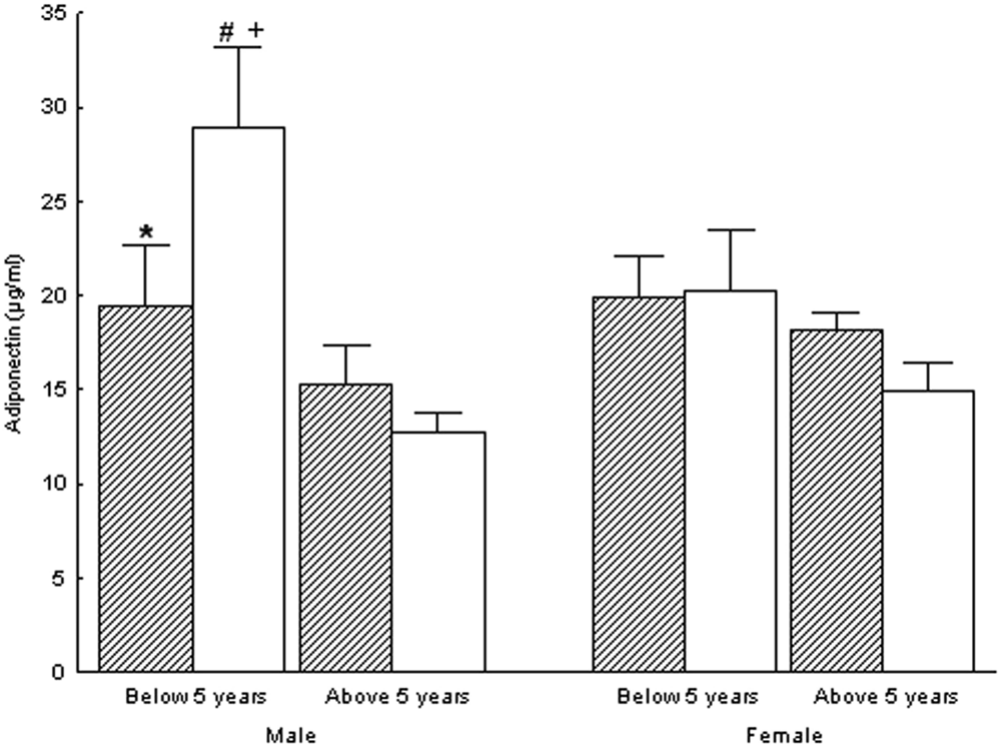
Adiponectin in the IUGR (hatched bars) and AGA (white bars) groups. Significant differences were assessed by One-Way factors ANOVA (p < 0.05). ^+^ denotes statistically significant difference from the respective female group. * denotes statistically significant difference from the respective AGA group. ^#^ denotes statistically significant difference from the respective group of more than 5 years old age.

Adipocytokines concentrations of the studied groups at the observation time are presented in Figs 2 and 3. Mean leptin concentration was significantly lower in male subjects born with IUGR and aged <5 years old than in male subjects born with IUGR and aged >5years old (p < 0.05), while the latter had significantly higher mean leptin concentration than in their respective control male subjects born AGA (p < 0.05). Mean adiponectin concentration was significantly lower in male subjects born with IUGR and aged <5 years old than in their respective control male subjects born AGA (p < 0.05). Male subjects born with IUGR and aged >5 years old had significantly higher mean leptin/waist and mean leptin/(waist/hip) ratios than their respective control male subjects born AGA (p < 0.05), while these parameters were significantly lower in female subjects born with IUGR and aged >5 years old than in their respective control female subjects born AGA (p < 0.05).

### Genetic Analysis of FTO rs9939609 polymorphism frequencies in children born with IUGR and in children born AGA

The frequencies of the genotypes of the *rs9939609* polymorphism of the FTO gene in the AGA and IUGR groups are presented in Table 6. When chi-square analysis followed by Fisher’s *post hoc* was applied, the frequencies of the genotypes did not differ statistically between the IUGR and AGA groups (p < 0.05). When the frequencies of the *rs9939609* polymorphism (T replaced by A) were tested for Hardy-Weinberg equilibrium, in the AGA and the IUGR groups, both were found to be in Hardy-Weinberg equilibrium (Table 7).

**Table 6:** Frequencies of the genotypes of the polymorphism in children born with IUGR compared to children born AGA, the x^2^ and corresponding P values for 2 degrees of freedom and the Freeman-Halton extension of the Fisher exact probability test, for two-rows by three-column contingency table.

Polymorphism	Genotype	AGA (n = 52)	IUGR (n = 45)	x^2^ (P)	Fischer’s (P_A_, P_B_, N. of tables)
*rs9939609*	**TT**	0.21 (n = 11)	0.33 (n = 15)	x^2^ = 2.6	**P**_**A**_ = 0.271
*rs9939609*	**TA**	0.56 (n = 29)	0.53 (n = 24)	P = 0.27	**P**_**B**_ = 0.271
*rs9939609*	**AA**	0.23 (n = 12)	0.13 (n = 6)		**N of tables evaluated** = 513

**Table 7:** Hardy-Weinberg Equilibrium analysis for children born IUGR and AGA.

	Observed	Expected	
**AGA**			
TT	11	12.5	**x**^**2**^ = 0.697
TA	29	26.0	
AA	12	13.5	**P** = 0.404 AGA in HWE
**IUGR**			
TT	15	16.2	**x**^**2**^ = 0.556
TA	24	21.6	
AA	6	7.2	**P** = 0.456 IUGR in HWE

When the FTO allele frequencies in the IUGR and AGA groups were calculated in a 2 × 2 contingency table no statistically significant difference was revealed between the two groups, for the T and A alleles. The frequencies of the genotypes of the *rs9939609* polymorphism were also tested for the presence of a dominant genetic model (A allele increases risk) in both IUGR and AGA groups. No statistically significant difference was revealed regarding the presence of the A allele. Finally, when the frequencies of the genotypes of the *rs9939609* polymorphism were tested for the presence of a recessive genetic model (two A alleles increase risk) in both IUGR and AGA groups, no statistically significant difference was found.

## Discussion

We found that birth weight and length were lower in all subjects born with IUGR compared to those born AGA, as expected^1,4^. Interestingly, males born with IUGR below 5 years old had higher weight z-score than their respective subjects born AGA, as well as their respective females born with IUGR. Of note, the latter did not differ in weight from their corresponding females born AGA, indicating that both males and females born with IUGR demonstrate weight catch-up before 5 years of age. However, in this study, males do so in a more prominent fashion. The preponderant expression of this weight catch-up in male children born with IUGR is not easily explainable. This observation should be included in the known “catch-up growth” phenomenon of infants born with IUGR. Furthermore, it should be corroborated with the known prevalence of metabolic syndrome observed in subjects born with IUGR in their adulthood^7,10^. When Milovanovic *et al*. compared children born SGA to children born AGA in their first and fourth year of age they found that the former experienced a greater weight catch-up during the first year of life, while at the age of 4 years their weight was lower than that of the children born AGA, albeit within normal range^36^. Previous studies suggested that rapid catch-up growth in early postnatal period was a risk factor for developing central fat mass and insulin resistance^37,38^. Babies born with IUGR usually present increased abdominal fat and increased biomarkers of insulin resistance despite the absence of difference in BMI as compared to children born AGA beyond 12 months of age^13^.

Regarding insulin secretion, in this study, all males born with IUGR as well as males born with IUGR older than 5 years old had higher C-peptide and insulin z-scores, respectively, than their respective control subjects born AGA. In addition, HOMA-IR index was found lower in males born with IUGR below 5 years old than in males born with IUGR above 5 years old. The hyperinsulinemia observed at an early age suggests that the insulin resistance developing in children born with IUGR during adulthood might have its origins even before childhood, *i.e. in utero*. In the past we have shown that during normal pregnancy in non-obese, non-diabetic women the increase of the adipose tissue from the first trimester of pregnancy is accompanied by a significant progressive increase of maternal insulin resistance^39^. Furthermore, in this study, leptin and adiponectin concentrations in males born with IUGR older and younger than 5 years old, respectively, were higher and lower, respectively, than their respective control AGA groups. Other authors reported lower concentrations of adiponectin in children born SGA as compared to children born AGA, while others suggested the development of leptin resistance *in utero*^40,41^. Previously, we measured these adipocytokines in the serum of mothers of babies born with IUGR and babies born AGA. The postnatal maternal concentrations of leptin and adiponectin were higher and lower, respectively, in the mothers of IUGR compared to those of AGA babies^42^. Thus, we suggested that children born with IUGR are associated to a specific high-leptin and low-adiponectin maternal profile. Also, in this study, leptin and adiponectin concentrations in male children born with IUGR were higher and lower, respectively, than their control AGA groups. Of note, leptin and adiponectin concentrations of male children born with IUGR, at the time of the study, were not statistically different from leptin and adiponectin concentrations of female children born with IUGR. It is possible that the small number of subjects included in this study did not allow the differences in leptin and adiponectin concentrations to appear as well between female children born with IUGR and those born AGA. The physiopathologic relationship of leptin with the development of insulin resistance together with the reported hyperleptinemia in children born with IUGR and their mothers suggest a possible genetic and/or epigenetic predisposition for the development of insulin resistance in children born with IUGR. In a rat model study the exposure to prenatal undernutrition led to the development of leptin resistance in adulthood, independently of diet-induced obesity^43^. These findings suggest that prenatal conditions such as undernutrition can lead to future changes in energy metabolism and development of metabolic syndrome in adulthood through alterations in leptin sensitivity *in utero*, while genetic predisposition in leptin production by adipose tissue might also contribute to this phenomenon. Other authors, though, reported no difference in leptin concentrations and higher adiponectin concentrations in children born SGA as compared to children born AGA^44^. The inclusion of children born SGA instead of those born with IUGR, as well as the differences in the number and age of the examined cases in that study compared to the present study might explain this discrepancy. Also, in that study no age group below 5 years old was included^44^.

Furthermore, the higher and lower concentrations of creatinine and eGFR, respectively, in all female subjects born with IUGR and the higher concentrations of creatinine in the younger than 5 years old female subjects born with IUGR as compared to their respective control AGA groups, might indicate that subjects born with IUGR might be at risk for developing renal derangement, particularly those of female sex. Previous studies reported reduced nephron number in children born with IUGR at birth^17^. A positive relationship between birth weight and the number of glomeruli, as well as a negative correlation between birth weight and glomerular volume in humans was also reported^18^.

Also, in this study, the higher concentrations of AST and ALT in the younger than 5 years old children born with IUGR than in their respective control subjects born AGA might indicate a developing liver dysfunction in subjects born with IUGR that starts in early childhood. In the past, liver biopsies associated IUGR with fatty liver in rats as well as with non-alcoholic fatty liver disease (NAFLD) in children^45,46^. It is possible that children born with IUGR might be predisposed to the development of liver dysfunction and NAFLD. The higher values of AST and ALT reported in this study might be associated to an early development of fatty liver in children born with IUGR. In addition, in this study, TG and uric acid z-scores were, respectively, higher in all females born with IUGR and in females born with IUGR older than 5 years old than in their respective control groups of children born AGA.

Regarding the frequency of the FTO gene *rs9939609* polymorphism we found no statistically significant difference among the groups of children born with IUGR and AGA. This polymorphism was in Hardy-Weinberg equilibrium in both groups.

In summary, we found that male children born with IUGR younger than 5 years old present with higher weight than their respective subjects born AGA. This finding associates enhanced weight increase to the “catch-up growth” phenomenon observed in children born with IUGR. Prepubertal boys born with IUGR, older and younger than 5 years old present, respectively, with increased concentrations of insulin and leptin and lower concentrations of adiponectin. This early observed hyperinsulinemia, hyperleptinemia and hypoadiponectinemia indicates that the known development of insulin and leptin resistance in adults born with IUGR manifests early in childhood. These findings should be corroborated to the increased prevalence of metabolic syndrome, including obesity, in adults born with IUGR. Creatinine is increased in all subjects born with IUGR, while hepatic enzymes AST and ALT are increased in all children born with IUGR younger than 5 years old and in male children born with IUGR younger than 5 years old, respectively. An adverse prenatal environment may induce early derangement of hepatic and renal function in IUGR fetuses, although the underlying mechanisms remain unclear.

In conclusion, this study highlights the importance of the early development of insulin and leptin resistance in children born with IUGR possibly associated to long-term metabolic consequences. Furthermore, it seems that there is an early development of hepatic and renal derangement in these children. More studies are needed to investigate the underlying mechanisms (possibly of epigenetic participation) which lead to changes in insulin and leptin sensitivity, as well as in derangements in the physiology of liver and kidneys. These events might occur *in utero* and in the early postnatal period. It is intriguing to investigate prospectively whether the suggested expression of features of the metabolic syndrome in these children during adulthood has its origins in prepubertal period.
